# Neutralizing activity of Sputnik V vaccine sera against SARS-CoV-2 variants

**DOI:** 10.21203/rs.3.rs-400230/v1

**Published:** 2021-04-08

**Authors:** Satoshi Ikegame, Mohammed N. A. Siddiquey, Chuan-Tien Hung, Griffin Haas, Luca Brambilla, Kasopefoluwa Y. Oguntuyo, Shreyas Kowdle, Ariel Esteban Vilardo, Alexis Edelstein, Claudia Perandones, Jeremy P. Kamil, Benhur Lee

**Affiliations:** 1. Department of Microbiology at the Icahn School of Medicine at Mount Sinai, New York, NY 10029, USA; 2. Department of Microbiology and Immunology, Louisiana State University Health Shreveport, Shreveport, LA 71103, USA.; 3. National Administration of Laboratories and Health Institutes of Argentina (ANLIS) Dr. Carlos G. Malbrán, Buenos Aires, Argentina

## Abstract

The novel pandemic betacoronavirus, severe acute respiratory syndrome coronavirus 2 (SARS-CoV-2), has infected at least 120 million people since its identification as the cause of a December 2019 viral pneumonia outbreak in Wuhan, China^1,2^. Despite the unprecedented pace of vaccine development, with six vaccines already in use worldwide, the emergence of SARS-CoV-2 ‘variants of concern’ (VOC) across diverse geographic locales have prompted re-evaluation of strategies to achieve universal vaccination^3^. All three officially designated VOC carry Spike (S) polymorphisms thought to enable escape from neutralizing antibodies elicited during initial waves of the pandemic^4–8^. Here, we characterize the biological consequences of the ensemble of S mutations present in VOC lineages B.1.1.7 (501Y.V1) and B.1.351 (501Y.V2). Using a replication-competent EGFP-reporter vesicular stomatitis virus (VSV) system, rcVSV-CoV2-S, which encodes S from SARS coronavirus 2 in place of VSV-G, and coupled with a clonal HEK-293T ACE2 TMPRSS2 cell line optimized for highly efficient S-mediated infection, we determined that only 1 out of 12 serum samples from a cohort of recipients of the Gamaleya Sputnik V Ad26 / Ad5 vaccine showed effective neutralization (IC_90_) of rcVSV-CoV2-S: B.1.351 at full serum strength. The same set of sera efficiently neutralized S from B.1.1.7 and showed only moderately reduced activity against S carrying the E484K substitution alone. Taken together, our data suggest that control of some emergent SARS-CoV-2 variants may benefit from updated vaccines.

SARS-CoV-2 is closely related to two other zoonotic betacoronaviruses, MERS-CoV and SARS-CoV, that also cause life-threatening respiratory infections ^9^. The global health emergency caused by the spread of SARS-CoV-2 has spurred the development of COVID-19 preventive vaccines at an unprecedented pace. Six are already authorized for human use across the globe ^10–15^. These vaccines focus on the SARS-CoV-2 spike protein (S), due to its critical roles in cell entry. Indeed, the presence of serum neutralizing antibodies directed at S correlate strongly with protection against COVID-19 ^16,17^. Although these six vaccines are efficacious, the recent emergence of novel SARS-CoV-2 variants has reignited concerns that the pandemic may not be so easily brought under control.

In December 2020, the United Kingdom reported the sudden emergence of a novel SARS-CoV-2 lineage, termed B.1.1.7 (501Y.V1, VOC 202012/01), which was designated as the first SARS-CoV-2 variant of concern (VOC). The lineage had rapidly increased in prevalence since first being detected in November 2020 ^18^. Its genome showed an unusually high number of non-synonymous substitutions and deletions, including eight in the S gene, suggesting a substantial degree of host adaptation that may have occurred during prolonged infection of an immunocompromised person ^19^. The B.1.1.7 lineage has now been shown to exhibit enhanced transmissibility ^20^ as well as an increased case fatality rate ^21,22^.

Soon afterwards, two additional SARS-CoV-2 VOC, B.1.351 and P.1, were reported from S. Africa and Brazil, respectively, which each showed substantial escape from neutralizing antibodies elicited by first wave pandemic viruses, leading to documented cases of re-infection ^23–25^. The S genes of B.1.351 and P.1 viruses each carry a number of mutations, but include three in the receptor binding domain (RBD) that are particularly notable, the S: N501Y substitution, found in B.1.1.7, alongside polymorphisms at positions 417 and 484, K417N/T and E484K. S: E484K had already been identified in multiple independent laboratories to confer escape from convalescent sera and monoclonal antibodies ^26–28^. As expected, the P.1 and B.1.351 variants escape or resist neutralization by first wave convalescent sera, as well as antibodies elicited by COVID-19 vaccines ^4–8^.

Although the P.1 and B.1.351 lineages are dominant in Brazil and S. Africa, unlike B.1.1.7 they have not increased greatly in number in the United States since originally being detected here. In contrast, the E484K polymorphism is recurrently emergent, and is found in a number of other lineages that are increasing in the U.S. and other countries. For example, a B.1.526 sub-lineage carrying E484K in recent weeks has expanded more rapidly than B.1.1.7 ^29,30^, which may be indicative of the ability of S: E484K variants to penetrate herd immunity. The P.2 lineage, originally detected in Rio de Janeiro, carries only the E484K mutation in the RBD and has spread to other parts of South America, including Argentina ^31^.

The six COVID-19 vaccines currently in use around the world employ different strategies, and do not all incorporate the two proline substitutions that “lock” S into the pre-fusion conformer. Vaccines that do not utilize pre-fusion “locked” S are expected to produce lower levels of neutralizing antibodies, and hence may be less efficacious against infection, even if they do protect against severe COVID-19. Indeed, a two-dose regimen of the AstraZeneca ChAdOx1 based vaccine, which does not use a “locked” S, did not protect against mild-to-moderate COVID-19 in S. Africa, where 93% of COVID-19 cases in trial participants were caused by the B.1.351 variant ^32^. Like the AstraZeneca ChAdOx1 vaccine, the Sputnik V vaccine (Gam-COVID-Vac) is based on adenovirus vectored expression of a native S sequence, rather than a pre-fusion “locked” S ^33^. Although the Sputnik V vaccine has a reported vaccine efficacy of 91.6% in the interim analysis of Phase 3 trials held in Russia between Sept 7 and Nov 24, 2020, none of the VOC mentioned above nor independent lineages containing the E484K mutation were prevalent in Russia during this time period. Since the Sputnik vaccine is now in use not only in Russia, but also in countries like Argentina, Mexico, and Hungary, where some of the VOC and emerging lineages bearing the E484K mutation are more widespread, it is critical to assess the neutralizing activity of Sputnik vaccine elicited antibody responses against these cognate VOC and mutant spikes.

This study characterizes the neutralization activity of sera from a dozen Sputnik V vaccine recipients in Argentina. Our work was spurred by Argentina’s nascent genomic surveillance efforts, which detected multiple independent lineages with S: E484K (B.1.1.318 and P.2) and/or S: N501Y substitutions (B.1.1.7 and P.1) in common, just as Argentina had started rolling out its vaccination campaign, which commenced on Dec 29, 2020. Here, we generated isogenic replication-competent vesicular stomatitis virus bearing the prevailing wild-type (WT=D614G) SARS-CoV-2 S (rcVSV-CoV2-S), or the B.1.1.7, B.1.351 or E484K mutant S and used them in a robust virus neutralization assay. Our results show that Sputnik V vaccine sera effectively neutralized S: WT and S: B.1.1.7. viruses, albeit with highly variable titers. The same sera, however, exhibited moderate and markedly reduced neutralization titers, respectively, against S: E484K and S: B.1.351. Analyses of dose response curves indicate that S: B.1.351 exhibits resistance to neutralizing sera in a manner that is qualitatively different from the E484K mutant. Taken together, our data argue that surveillance of the neutralizing activity elicited by vaccine sera will be necessary on an ongoing basis. Viral neutralization assays can indicate which SARS-CoV-2 variants are likely capable of transmission in the face of vaccine elicited immunity, and whether updated vaccines will be needed to control their emergence and spread.

## RESULTS.

### Robust reverse genetics for generating replication-competent VSV expressing SARS-CoV-2 Spike proteins.

Several groups have now generated replication-competent VSV expressing SARS-CoV-2 spike in place of VSV-G (rcVSV-CoV2-S)^34–36,37^. These rcVSV-CoV2-S can be used in BSL-2 compatible virus neutralization assays (VNAs), which correlate very well with VNAs using live SARS-CoV-2 (Spearman’s r > 0.9 across multiple studies). rcVSV-CoV2-S has been assessed as a candidate vaccine ^36,38^, and used in forward genetics experiments to generate antibody escape mutants or perform comprehensive epitope mapping studies ^39,26,37^. Indeed, the now concerning E484K mutation, present in many variants of concern (VOC), was identified as an antibody escape mutation using rcVSV-CoV-2-S ^26,37^.

However, many groups passage their rcVSV-CoV-2-S extensively in Vero cells after the initial rescue, either to generate higher titer stocks and/or to remove confounding components such as the vaccinia virus expressing T7-polymerase and/or transfected VSV-G, both of which were deemed necessary for efficient rescue ^37^. Serial passage of rcVSV-CoV-2-S in Vero cells invariably leads to mutations in the S1/S2 furin cleavage site, as well as truncations in the cytoplasmic tail of the S protein ^38^. The latter promotes S incorporation into VSV without compromising the conformational integrity of the ectodomain, whereas the former is problematic when assessing the neutralization sensitivity and structure-function phenotype of Spike VOC with multiple mutations that likely have complex epistatic interactions.

**Figure 1.**
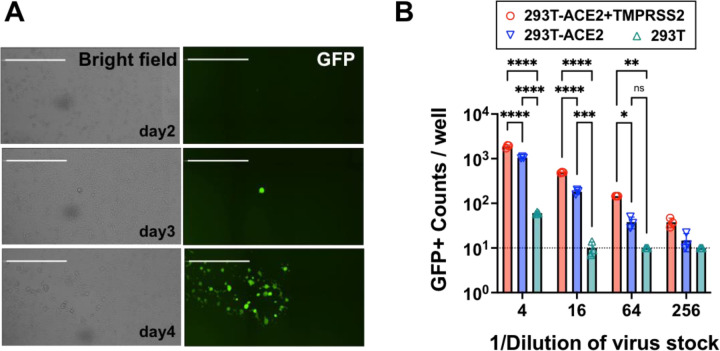
Generation of replication-competent VSV bearing SARS-CoV-2 spike (rcVSV-CoV2-S). **(A)** Representative images of *de novo* generation of rcVSV-CoV2-S, carrying an EGFP reporter, in transfected 293T-ACE2+TMPRSS2 (F8–2) cells as described in Extended Data Fig. S1. Single GFP+ cells detected at 2–3 days post-transfection (dpt) form a foci of syncytia by 4 dpt. Images are taken by Celigo imaging cytometer (Nexcelom) and are computational composites from the identical number of fields in each well. White bar is equal to 1 millimeter. **(B)** Entry efficiency of rcVSV-CoV2-S in parental 293T cells, 293T stably expressing ACE2 alone (293T-ACE2) or with TMPRSS2 (293T-ACE2+TMPRSS2). Serial dilutions of virus stocks amplified on Vero-TMPRSS2 cells were used to infect the indicated cell lines in 96-well plates in triplicates. GFP signal was detected and counted by a Celigo imaging cytometer (Nexcelom) 10 hours post-infection. Symbols are individual data points from triplicate infections at the indicated dilutions. Bars represent the average of 3 replicates with error bars indicating standard deviation. A two-way ANOVA was used to compare the differences between cell lines at any given dilution. Adjusted p values from Tukey’s multiple comparisons test are given (ns; not significant, * p < 0.05, ** p < 0.01, *** p < 0.001, **** p < 0.0001).

To generate rcVSV-CoV2-S containing different variants or mutants on demand, without the need for extensive passaging, we developed a robust reverse genetics system and VNA which leverages the cell lines we previously developed for a standardized SARS-CoV-2 VNA that correlates well with live virus neutralization ^40^. Salient improvements include the addition of a hammerhead ribozyme immediately upstream of the 3’ leader sequence which cleaves *in cis* to give the exact 3’ termini, the use of a codon-optimized T7-polymerase which alleviates the use of vaccinia-driven T7-polymerase, and a highly permissive and transfectable 293T-ACE2+TMPRSS2 clone (F8–2) ^40^ (Extended Data Fig S1). A 6-plasmid transfection into F8–2 cells results in GFP+ cells 2–3 days post-transfection (dpt), which turn into foci of syncytia by 4–5 dpt indicating virus replication and cell-to-cell spread (Fig. 1A). Transfer of F8–2 cell supernatant into interferon-defective Vero-TMPRSS2 cells allowed for rapid expansion of low-passage viral stocks that maintain only the engineered Spike mutations. Clarified viral supernatants from Vero-TMPRSS2 cells were aliquoted, sequenced verified, then titered on F8–2 cells to determine the linear range of response (Fig. 1B).

**Figure 2.**
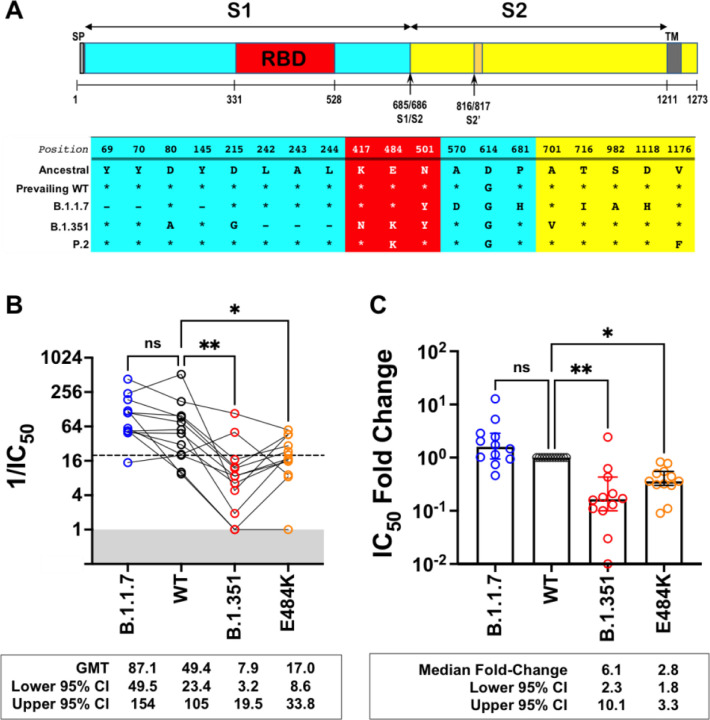
Neutralization activity of antibody responses elicited by the Sputnik V vaccine. **(A)** Schematic of the Spike substitutions that make up the variants being evaluated in this study. The amino acid positions and corresponding ‘Ancestral’ sequence of the Wuhan isolate is shown. The prevailing WT sequence now has a D614G substitution. All the variants and mutants have D614G. **(B)** Neutralization activity of individual serum samples against rcVSV-CoV2-S with the WT, variant (B.1.1.7 or B.1.351), or mutant E484K spike proteins. Neutralization is represented by the reciprocal 50% inhibitory dilution factor (1/IC_50_). Sera samples with no appreciable neutralization against a given virus were assigned a defined 1/IC_50_ value of 1.0, as values ≤1 are not physiological (Grey shaded area). Dashed line indicates the lowest serum dilution tested (1/IC50 = 20). Geometric mean titers (GMT and 95% CI) for the neutralizing activity of all vaccine sera are indicated below each of the viral spike proteins examined. NS; not significant, *; p<0.05, p < 0.01; ** are adjusted p values from non-parametric one-way ANOVA with Dunn’s multiple comparisons test. **(C)** For each serum sample, the fold-change in IC_50_ (reciprocal inhibitory dilution factor) against the indicated variant and mutant spike proteins relative to its IC_50_ against wild-type (WT) spike (set at 1) is plotted. Adjusted p values were calculated as in (B). Medians are represented by the bars and whiskers demarcate the 95% CI. Neutralization dose-response curves were performed in triplicates, and the mean values from each triplicate experiment are shown as the single data points for each sera sample.

Next, we generated isogenic rcVSV-CoV2-S expressing the B.1.1.7, B.1.351 (Fig. 2A), or E484K S to evaluate the neutralizing activity of Sputnik V vaccine sera from Argentina. The relevant Spike substitutions that make up these variants are indicated in Fig. 2A. The characteristics of the vaccine recipient cohort (n=12) receiving the two-dose regimen of the Sputnik vaccine are given in Table 1. At one month post-completion of the two-dose regimen, the Sputnik V vaccine generated respectable virus neutralizing titers (VNT) against rcVSV-CoV2-S bearing the WT (D614G) and B.1.1.7 spike proteins (Fig. 2B). The geometric mean titer (GMT) and 95% CI for WT (1/IC_50_ GMT 49.4, 23.4 – 105) in our cohort of vaccine recipients was remarkably similar to that reported in the phase III Sputnik vaccine trial (GMT 44.5, 31.8 – 62.2)^10^. However, GMT against B.1.351 and E484K was reduced by a median 6.8- and 2.8-fold, respectively compared to WT (Fig. 2C).

**Figure 3.**
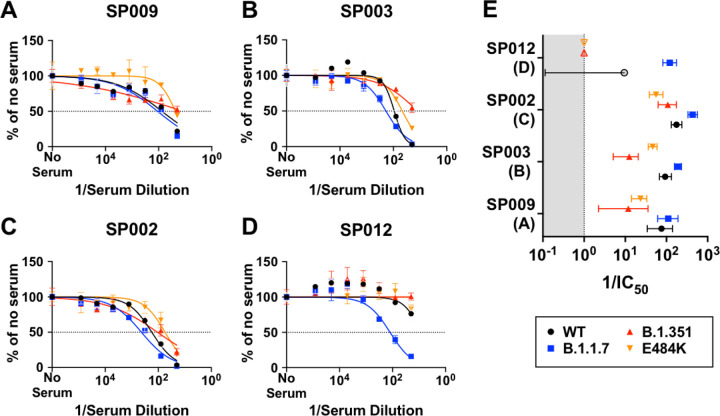
Dose response curves reveal distinct patterns of neutralizing antibody responses. Groups **(A - D)** represent distinct classes of virus neutralizing activity present in the sera samples analyzed. A representative member from each group is shown. Full neutralization curves for all sera tested against all viruses bearing the variant and mutant spike proteins are shown in supplementary Fig. S2. **(E)** graphs the virus neutralizing titers (VNT = 1/IC_50_) and 95% CI that can be extrapolated from the nonlinear regression curves. Different colored symbols represent the viruses indicated in the figure key. The open symbols in SP012 (Group D) represent assigned values of 1.0 (for B.1.351 and E484K) when no significant neutralization activity could be detected at the lowest serum dilution used (1:20) or ambiguous fits (for WT) due to very low neutralizing activity. The shaded area represents values that are not physiologically relevant.

Sputnik vaccine recipients appeared to generate qualitatively different neutralizing antibody responses against SARS-CoV-2 (Fig.3A−D and Extended Data Fig. S2) that segregated into 4 different groups. Group (A) exemplified by SP009 showed reasonable VNT against wild-type (WT) and B.1.1.7 (reciprocal IC_50_ = 76 and 111, respectively, Fig. 3A and E). However, the Hill slope of the neutralization curve for B.1.351 was extremely shallow (h=0.39). This class of sera achieves a maximal neutralization of 50–60% even when extrapolated to full serum strength (1/serum dilution = 1). In contrast, although the reciprocal IC_50_ for E484K is moderately decreased (VNT = 23), it is clear that E484K will still be neutralized at higher serum concentrations due to a significantly steeper Hill slope (h=1.4). Group (B) sera generally exhibit effective neutralization of WT, B.1.1.7, and even E484K at high serum concentrations, but not B.1.351 (Fig. 3B and E). The decreased potency and shallow Hill Slope result in <90% neutralization of B.1.351 even at full serum strength. Group (C) sera neutralize E484K and B.351 with potencies similar to WT and B.1.1.7, especially at high serum concentrations (Fig. 3C and E). This group of sera reveals that qualitatively different neutralizing responses can be generated that can effectively neutralize B.1.351. The one serum in Group (D) appears unique. It exhibited little to no neutralizing activity against WT, E484K and B.1.351, yet it neutralized B.1.1.7 as well as Group A-C sera. This is obvious when comparing the reciprocal IC50s for B.1.1.7 (blue squares) across the four different groups of sera in Fig.3D and E).

**Figure 4.**
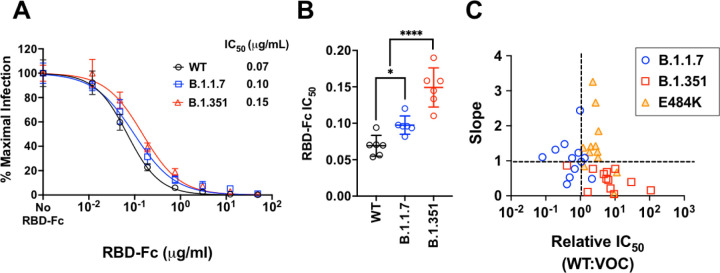
Competitive inhibition of rcVSV-CoV2-S entry by soluble RBD-Fc. **(A)** Recombinant RBD-Fc was serially titrated with the infection inoculum containing a fixed amount of rcVSV-CoV2-S bearing WT or the indicated VOC spike proteins. 10 hpi, GFP+ cells were quantified by the Celigo image cytometer. Data points are means of six independent replicates with error bars representing S.D. The number of GFP+ cells in the absence of any RBD-Fc was set to 100% and used to normalize the infection response in the presence of increasing amounts of RBD-Fc. Log[inhibitor] versus normalized response variable slope nonlinear regression curves were generated using GraphPad PRISM (v9.1.0). **(B)** The IC50 values from each replicate dose response curve generated for a given virus were grouped. The mean (central bar) and SD (whiskers) for each group are indicated. Adjusted p values (*, p<0.05; ****, p<0.0001) from ordinary one-way ANOVA with Dunnett’s multiple comparisons test are indicated. **(C)** Landscape of slope versus relative IC50 values of all 12 Sputnik sera against the indicated VOC or mutant Spike. Relative IC_50_ value is defined as the ratio of WT IC_50_:VOC IC_50_. Dashed lines indicate quadrants of high/low IC_50_ and high/low slope.

The heterogenous dose-response curves described in Fig. 3 (and Extended Data Fig. S2) is a property of Sputnik V vaccine elicited responses as soluble RBD-Fc inhibition of WT and VOC S-mediated entry produced classical dose response curves with Hill slopes close to −1.0 (Fig. 4A). Both B.1.1.7 and B.1.351 were modestly but significantly more resistant to RBD-Fc inhibition (Fig. 4B). This is not surprising as both harbor the N501Y mutation known to enhance affinity of RBD for ACE2. However, this 1.5 to 2-fold increase in RBD-Fc IC_50_ for B.1.1.7 and B.1.351, respectively, does not explain the neutralization-resistant versus sensitive phenotype of B.1.351 versus B.1.1.7 in our virus neutralization assays. Interestingly, each VOC or mutant clustered differently in the neutralization phenotype landscape defined by both IC50 and slope (Fig. 4C).

These data suggest that the cognate VOC exhibit qualitatively distinct modes of escape from Sputnik vaccine neutralization ^41^.

## DISCUSSION

A key public health concern related to emergent SARS-CoV-2 variants is that by incrementally accruing mutations that escape neutralizing antibodies, they will penetrate herd immunity and spread to reach unvaccinated individuals, some of whom will be susceptible to severe or fatal disease.

Three of the six COVID-19 vaccines currently in use worldwide, namely Moderna mRNA-1273, BioNTech BNT162b2, and Janssen Ad26.COV2.S, each express S harboring K986P and V987P substitutions (2P) within a loop abutting the central helix of the S2’ membrane fusion machinery ^42–44^. This modification locks the spike in a prefusion conformation and elicits higher titers of neutralizing antibodies ^45,46^. Of the three vaccines that do not appear to make use of 2P Spike mutants, Gamaleya’s Sputnik V and AstraZeneca’s AZD1222 are adenovirus-vectored vaccines encoding native S. The third is CoronaVac, a preparation of inactivated SARS-CoV-2 virions. Although all six vaccines are highly efficacious at preventing severe COVID-19 outcomes, they do not all uniformly prevent infection. Moreover, in all cases thus far examined, these first generation vaccines are less effective against variants with certain non-synonymous substitutions in Spike, such as E484K.

The most concerning variants are those with multiple mutations in the receptor binding domain (RBD) that confer both enhanced affinity for the hACE2 receptor and escape from neutralizing antibody responses ^5,8,23,32,47,48^. B.1.351 and P.1 have in common three RBD substitutions (K417N/T, E484K and N501Y) whereas all three WHO designated VOC contain the N501Y substitution. Although B.1.1.7 shows enhanced transmissibility and more severe disease outcomes^20,21^, it does not appear to be consistently more resistant to serum neutralizing responses elicited by vaccines or natural infection ^49,50^. The same is not true, however, for the B.1.351 variant.

In live virus plaque reduction neutralization assays, sera from AstraZeneca vaccine recipients in South Africa exhibited 4.1 to 32.5-fold reduction in neutralizing activity against B.1.351 ^32^. The actual reduction is even more marked because 7 of 12 vaccine recipients who had neutralizing activity against the parental B.1.1 variant, had undetectable neutralization against the B.1.351 strain. Comparator sera from recipients of Moderna and BioNTech mRNA vaccines showed smaller, 6.5 to 8.6-fold reductions in neutralization ^51^.

As of this writing, there is no data on the protective efficacy of Sputnik V and CoronaVac against SARS-CoV-2 S variants. Here, we showed that sera from Sputnik vaccine recipients in Argentina had a median 6.1-fold and 2.8-fold reduction in GMT against B.1.351 and the E484K mutant spike, respectively. Even more revealing is their dose-response curves. When extrapolated to full serum strength, half of the sera samples failed to achieve an IC80 and only 1 out 12 achieved an IC_90_. (Extended Data Fig. S3). One serum had little to no detectable neutralizing activity against B.1.351, E484K and even WT, but neutralized B.1.1.7 effectively. Altogether, these data suggest vaccines that do not use the 2P stabilized Spike appear to generate more variable neutralizing responses that make it difficult to establish immune correlates of protection, especially against emerging VOC/VOI that contain the recurrent E484K mutation.

E484K is present not only as part of an ensemble of RBD mutations present in B.1.351 and P.1, but in many of the 17 lineages detected from South America that carry it, such as P.2, E484K is the only RBD substitution (Supplementary Table 1). A more detailed report covering the genomic surveillance efforts in Argentina that detected the VOC which spurred our study is currently in preparation (Dr. Claudia Perandones, personal communication).

While the E484K substitution appears to be a common route of escape from many RBD-targeting monoclonal antibodies, it is somewhat surprising that a single mutation can confer a significant degree of neutralization resistance from polyclonal responses. Nonetheless, our data show that resistance conferred by E484K mutation be overcome by higher titer antibodies present in undiluted patient sera. But the neutralization resistance conferred by the suite of mutations present in B.1.351 appears qualitatively different. In the majority of cases, the slope of the dose response curve indicates a failure to neutralize even at full strength. We had previously shown that the dose-response curve slope is a major predictor of therapeutic potency for HIV broadly neutralizing antibodies at clinically relevant concentrations ^41^. Importantly, the slope parameter is independent of IC50 but is specifically related to an antibody’s epitope class. Here, we show that defining the neutralization phenotype of a given spike variant or mutant by both its relative IC50 and slope provides a fuller characterization of serum neutralizing activity against SARS-CoV-2 spike and the emergent VOC.

Although we stress that the Gameyla Sputnik V vaccine is likely to retain strong efficacy at preventing severe COVID-19, even in the case of infection by VOC, our data reveal a concerning potential of B.1.351, and to a lesser extent, any variant carrying the E484K substitution (e.g. P.2), to escape the neutralizing antibody responses that this immunization elicits. Furthermore, we acknowledge that *in vivo* protective efficacy can be derived from Fc effector functions of antibodies that bind but do not neutralize. In addition, an adenoviral vectored vaccine should induce potent cell-mediated immunity against multiple epitopes, which were not measured in our study. Nevertheless, given the crucial roles neutralizing antibodies play in preventing infection, our results suggest that updated SARS-CoV-2 vaccines will be necessary to eliminate the virus.

## Materials and Methods

### Cell lines

Vero-CCL81 TMPRSS2, HEK 293T-hACE2 (clone 5–7), and 293T-hACE2-TMPRSS2 (clone F8–2) cells were described previously ^40^, and were maintained in DMEM + 10%FBS. The HEK 293T-hACE2-TMPRSS2 cells were plated on collagen coated plates or dishes. BSR-T7 cells ^52^, which stably express T7-polymerase were maintained in DMEM with 10% FBS.

### VSV-eGFP-CoV2 spike (∆21aa) genomic clone and helper plasmids.

We cloned VSV-eGFP sequence into pEMC vector (pEMC-VSV-eGFP), which includes an optimized T7 promoter and hammerhead ribozyme just before the 5’ end of the viral genome. The original VSV-eGFP sequence was from pVSV-eGFP, a generous gift of Dr. John Rose ^53^.

We generated pEMC-VSV-eGFP-CoV2-S (Genbank Accession: MW816496) as follows: the VSV-G open reading frame of pEMC-VSV-eGFP was replaced with the SARS-CoV-2 S, truncated to lack the final 21 amino acids ^54^. We introduced a Pac-I restriction enzyme site just after the open reading frame of S transcriptional unit, such that the S transcriptional unit is flanked by MluI / PacI sites. SARS-CoV-2 S is from pCAGGS-CoV-2-S ^55^, which codes the codon optimized S from the Wuhan Hu-1 isolate (NCBI ref. seq. NC_045512.2) with a point mutation of D614G, resulting in B.1 lineage. The B.1.1.7 Spike we used carries the mutations found in GISAID Accession Number EPI_ISL 668152: del 69–70, del145, N501Y, A570D, D614G, P681H, T716I, S982A, and D1118H. The B.1.351 Spike carries the mutations D80A, D215G, del242–244, K417N, E484K, N501Y, D614G, and A701V (from EPI_ISL_745109). The Spike sequences of WT, B.1.1.7, B.1.351, and E484K are available at Genbank (Accession Numbers: MW816497, MW816498, MW816499, and MW816500; please also see Supplemental Table 2).

Sequences encoding the VSV N, P, M, G, and L proteins were also cloned into pCI vector to make expression plasmids for virus rescue, resulting in plasmids: pCI-VSV-N, pCI-VSV-P, pCI-VSV-M, pCI-VSV-G, and pCI-VSV-L. These accessory plasmids were a kind gift from Dr. Benjamin tenOever.

### Generation of VSV-CoV2 spike from cDNA

4 × 10^5^ 293T-ACE2-TMPRSS2 cells per well were seeded onto collagen-I coated 6 well plates. The next day, 2000 ng of pEMC-VSV-EGFP-CoV2 spike, 2500 ng of pCAGGS-T7opt ^56^, 850 ng of pCI-VSV-N, 400 ng of pCI-VSV-P, 100 ng of pCI-VSV-M, 100 ng of pCI-VSV-G, 100 ng of pCI-VSV-L were mixed with 4 mL of Plus reagent and 6.6 mL of Lipofectamine LTX (Invitrogen). 30 min later, transfection mixture was applied to 293T-hACE2-TMPRSS2 cells in a dropwise fashion. Cells were maintained with medium replacement every day for 4 to 5 days until GFP positive syncytia appeared. Rescued viruses were amplified in Vero-CCL81 TMPRSS2 cells ^40^, then titered and used for the assay.

### Virus neutralization assay

5 x 10E4 293T-hACE2-TMPRSS2 cells per well were seeded onto collagen-coated 96 well cluster plates one day prior to use in viral neutralization assays. Virus stocks were mixed with serially diluted serum for 10 minutes at room temperature, then infected to cells. Note: all sera assayed in this study were previously heat inactivated by 56 degrees for 30 min before use in any viral neutralization studies. At 10 h post infection, GFP counts were counted by Celigo imaging cytometer (Nexcelom). Each assay was done in triplicate. For calculation of IC50, GFP counts from “no serum” conditions were set to 100%; GFP counts of each condition (serum treated) were normalized to no serum control well. Inhibition curves were generated using Prism 8.4.3 (GraphPad Software) with ‘log (inhibitor) vs normalized response - variable slope’ settings.

### Design of RBD-Fc producing Sendai virus

Sendai virus (SeV) Z strain cDNA sequence (AB855655.1) was generated and cloned into pRS vector with the addition of eGFP transcriptional unit at the head of SeV genome. The sequence of F transcriptional unit was from SeV fushimi strain (KY295909.1) due to the cloning reason. We refer to the pRS-based plasmid coding this sequence as pRS-SeVZ-GFP-F^fushimi^ in this paper. For the introduction of foreign gene into SeV, we generated additional transcriptional unit for RBD-Fc between P gene and M gene. RBD-Fc construct was generated as below; codon optimized DNA sequence of from SARS-CoV-2 spike (MN908947) in pCAGGS a gift of Dr. Florian Krammer ^55^. S amino acids 319 – 541 (corresponding to the RBD domain) sequence were C-terminally fused to the Fc region of human IgG1 (220 – 449 aa of P0DOX5.2)

### Generation of recombinant Sendai virus from cDNA.

2x10E5 BSR-T7 cells per well were seeded onto 6-well cluster plates. The next day, 4 µg of pRS-SeVZ-GFP-F^fushimi^, 4 µg of pCAGGS-T7opt, 1.44 µg of SeV-N, 0.77 ug of SeV-P, 0.07 ug of SeV-L were mixed with 5.5 µl of Plus reagent and 8.9 µl of Lipofectamine LTX (Invitrogen). 30 min later, transfection mixtures were applied to Bsr-T7 cells in a dropwise fashion, as described previously ^56^. At one day post transfection, medium was replaced with DMEM + 0.2 µg/ml of TPCK-trypsin (Millipore Sigma, #T1426), with subsequent medium replacement each day until infection reached 100% cytopathic effect. Supernatants were stored at −80°C until use in experiments.

### Titration of viruses.

For SeV titration, 2 x 10E4 Bsr-T7 cells per well were seeded onto 96-well plates. The next day, 100 µL of serially diluted virus stock (in DMEM + 10% FBS) were applied to each well. GFP positive foci were counted at 24 hours post infection using a Celigo imaging cytometer (Nexcelom, Inc.). Infectivity is presented in infectious units (IU) per mL.

For VSV-CoV2 titration, 5 x 10E4 293T-hACE2-TMPRSS2 cells per well were seeded onto a collagen-coated 96 well plate. Serially diluted virus stocks were then applied to the cells, and GFP positivity was scored at 10 h post infection using a Celigo imaging cytometer.

### Production of proteins and purification.

5×10E6 Bsr-T7 cells are seeded in T175cm^2^-flask one day before infection. Cells were infected by SeV at MOI of 0.1 for one hour, followed by replacement of medium with DMEM supplemented with 0.2 mg/mL TPCK-trypsin. Medium was replaced with fresh 0.2 mg/ml TPCK-trypsin containing DMEM each day until infection reached 100% CPE, at which point medium was exchanged for DMEM lacking TPCK-trypsin. Cells were incubated for additional 24 h to allow protein production. Supernatants were centrifuged at 360 *g* for 5 min, then filtered with 0.1 µm filter (Corning® 500 mL Vacuum Filter/Storage Bottle System, 0.1 µm Pore) to remove virions and debris. Supernatant including RBD-Fc were applied to Protein G Sepharose (Millipore Sigma, #GE17–0618-01) containing column (5ml Polypropylene Columns; ThermoFisher, #29922), followed by wash and elution.

### Human Subjects Research.

Human subjects research was conducted following the Declaration of Helsinki and related institutional and local regulations. Studies and serum collection relating to the Sputnik vaccine at ANLIS Dr. Carlos G. Malbrán (NatIonal Administration Laboratories and Health Institutes - Carlos G. Malbrán, Argentina) were approved by the Research Ethics Committee of its Unidad Operativa Centro de Contención Biológica (UOCCB) on 9 Feb 2021.

## Supplementary Material

Supplement SUPPLEMENTAL TABLE 1. Acknowledgement of S: E484K viruses from South America shared on GISAID.

Supplement SUPPLEMENTAL TABLE 2. Acknowledgement of B.1.1.7 and B.1.351 viruses used for selection of S variants evaluated in this study.

Supplement

Supplement

Supplement 5

## Figures and Tables

**Table 1.** Cohort characteristics of Sputnik vaccine recipients from ANLIS MALBRÁN (Buenos Aires, República Argentina).

Sera ID	1^st^ DOSE	2^nd^ DOSE	Vaccine Status	SEX	AGE
SP001	Late Dec/2020	Mid Jan/2021	(+)	M	45–50
SP002	Late Dec/2020	Mid Jan/2021	(+)	M	40–45
SP003	Late Dec/2020	Mid Jan/2021	(+)	M	55–60
SP004	Late Dec/2020	Mid Jan/2021	(+)	M	50–55
SP005	Late Dec/2020	Mid Jan/2021	(+)	M	35–40
SP006	Late Dec/2020	Mid Jan/2021	(+)	F	35–40
SP007	Late Dec/2020	Mid Jan/2021	(+)	F	20–25
SP008	Late Dec/2020	Early Feb/2021	(+)	M	35–40
SP009	Late Dec/2020	Early Feb/2021	(+)	F	30–35
SP010	Late Dec/2020	Mid Jan/2021	(+)	M	30–35
SP011	Late Dec/2020	Mid Jan/2021	(+)	M	40–45
SP012	Late Dec/2020	Mid Jan/2021	(+)	M	25–30
				**Median Age**	**39.5**
				**Range**	**25–56**
SP013	N.A.	N.A.	(−)	F	45–50
SP014	N.A.	N.A.	(−)	F	50–55
SP015	N.A.	N.A.	(−)	M	40–45

N.A., Not Applicable
