# Supplementary material for: Neutralizing activity of Sputnik V vaccine sera against SARS-CoV-2 variants
Source: Res Sq. 2021 Apr 8:rs.3.rs-400230. Preprint. [Version 1] doi: 10.21203/rs.3.rs-400230/v1 (PMC8043464; doi:10.21203/rs.3.rs-400230/v1)
Supplement: Supplement — AL TABLE 1. Acknowledgement of S: E484K viruses from South America shared on GISAID. [file 4bbd1cf6796443968aa930d6.pdf]

Accession ID	Virus Name	Spike polymorphisms	PANGO Lineage	Originating Laboratory	Submitting Laboratory	Authors
EPI_ISL_745109	hCoV-19/South Africa/Tygerberg-419/202	H69del, V70del, Y144del, N501Y, A570D, D614G, P681H, D1118H, S982A, T716I,	B.1.351	Kraaifontein CHC wc KFP, Division of Medical Virology, Tygerberg Academic Hospital, Tygerberg 7505, Western Cape, South Africa	National Health Laboratory Service (NHLS), Tygerberg, Division of Medical Virology, Tygerberg Academic Hospital, Tygerberg 7505, Western Cape, South Africa	Susan Engelbrecht, Kayla Delaney, Bronwyn Kleinhans, Houriiyah Tegally, Eduan Wilkindon, Gert van Zyl, Wolfgang Preiser, Tulio de Oliveira
EPI_ISL_668152	hCoV-19/England/QEUH-B813E5/2020	D80A, D215G, L242del, A243del, L244del, K417N, E484K, N501Y, D614G, A701V	B.1.1.7	Lighthouse Lab in Glasgow, Teaching & Learning Centre, Queen Elizabeth University Hospital campus, 1345 Govan Road, Glasgow, G51 4TF, UK	Wellcome Sanger Institute for the COVID-19 Genomics UK (COG-UK) Consortium, Wellcome Genome Campus, Hinxton, Cambridgeshire, UK, CB10 1SA	Harper VanSteenhouse, Yumi Kasai, David Gray, Carol Clugston, Anna Dominiczak and Alex Alderton, Roberto Amato, Sonia Goncalves, Ewan Harrison, David K. Jackson, Ian Johnston, Dominic Kwiatkowski, Cordelia Langford, John Sillitoe on behalf of the Wellcome Sanger Institute COVID-19 Surveillance Team
