# Supplementary material for: Neutralizing activity of Sputnik V vaccine sera against SARS-CoV-2 variants
Source: Res Sq. 2021 Apr 8:rs.3.rs-400230. Preprint. [Version 1] doi: 10.21203/rs.3.rs-400230/v1 (PMC8043464; doi:10.21203/rs.3.rs-400230/v1)
Supplement: Supplement 5 [file NIHPPRS400230v1-supplement-5.pdf]

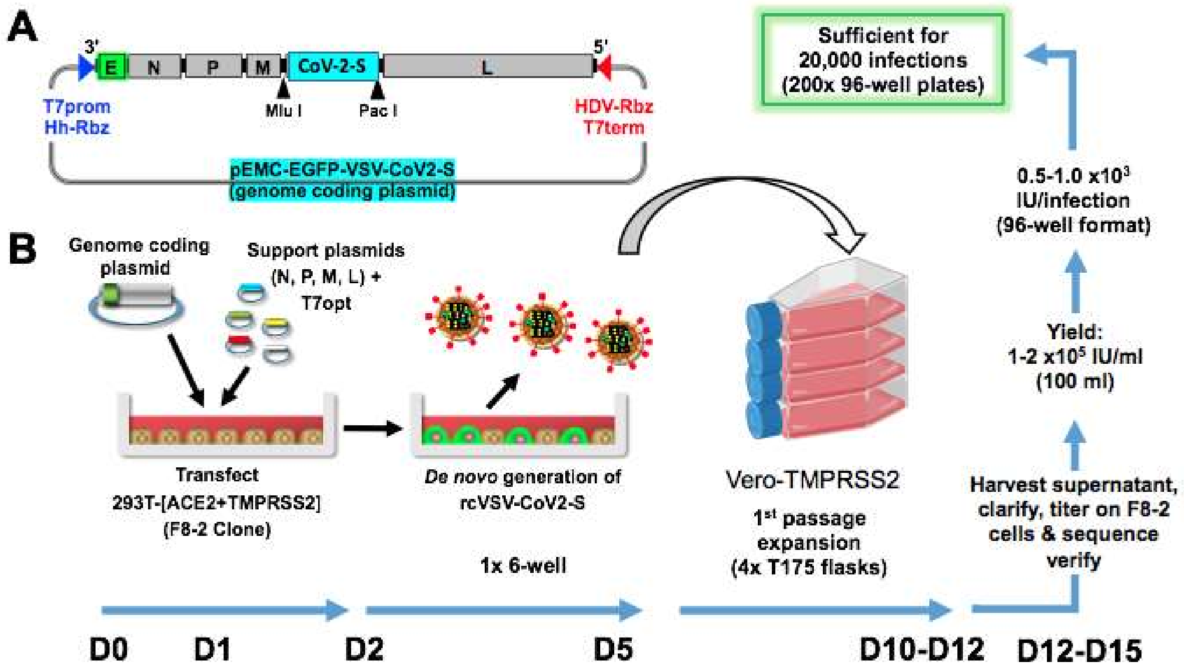

Extended Data Figure S1. Robust and efficient generation of an EGFP-reporter replication-competent VSV bearing SARS-CoV-2 spike (rcVSV-CoV2-S). (A) Schematic of the rcVSV-CoV2-S genomic coding construct and the virus rescue procedure. The maximal T7 promoter (T7prom) followed by a hammer-head ribozyme (HhRbz) and the HDV ribozyme (HDVRbz) plus T7 terminator (T7term) are positioned at the 3' and 5' ends of the viral cDNA, respectively. An EGFP(E) transcriptional unit is placed at the 3' terminus to allow for high level transcription. SARS-CoV-2-S is cloned in place of VSV-G using the indicated restriction sites designed to facilitate easy exchange of spike variant or mutants. (**B)** For virus rescue, highly permissive 293T cells stably expressing human ACE2 and TMPRSS2 (293T-[ACE2+TMPRSS2], F8-2 clone) cells were transfected with the genome coding plasmid, helper plasmids encoding CMV-driven N, P, M, and L genes, and pCAGS encoding codon-optimized T7-RNA polymerase(T7opt). 48-72 hpi, transfected cells turn EGFP+ and start forming syncytia. Supernatant containing rcVSV-CoV2-S are then amplified in Vero-TMPRSS2 cells at the scale shown. The blue arrows at the bottom indicate the timeline for production of each sequence verified stock.

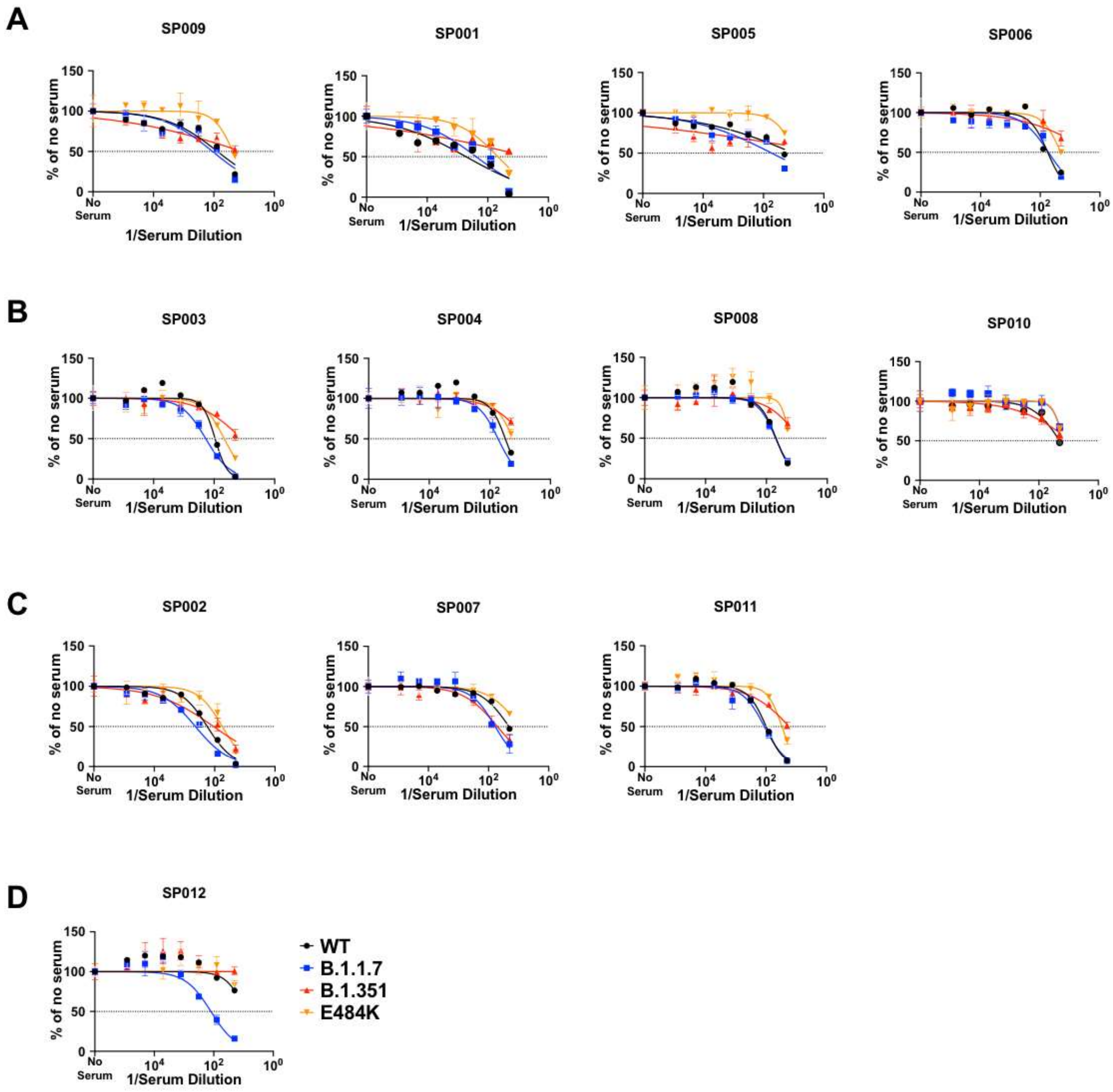

Extended Data Fig. S2. Sputnik vaccine elicits qualitatively different polyclonal responses against SARS-CoV-2 Spike. The full neutralization dose-response curves for all four classes of sera described in Fig. 3 are shown. Data points are mean of 3 independent replicates with error bars representing S.D. Infection (GFP+ cells) at each serum dilution was normalized to that obtained in the absence of any serum (set at 100%). Nonlinear regression of log [reciprocal serum dilution] versus normalized infection was performed using GraphPad PRISM (v9.0.1). Dotted lines represent 50% of maximal infection.

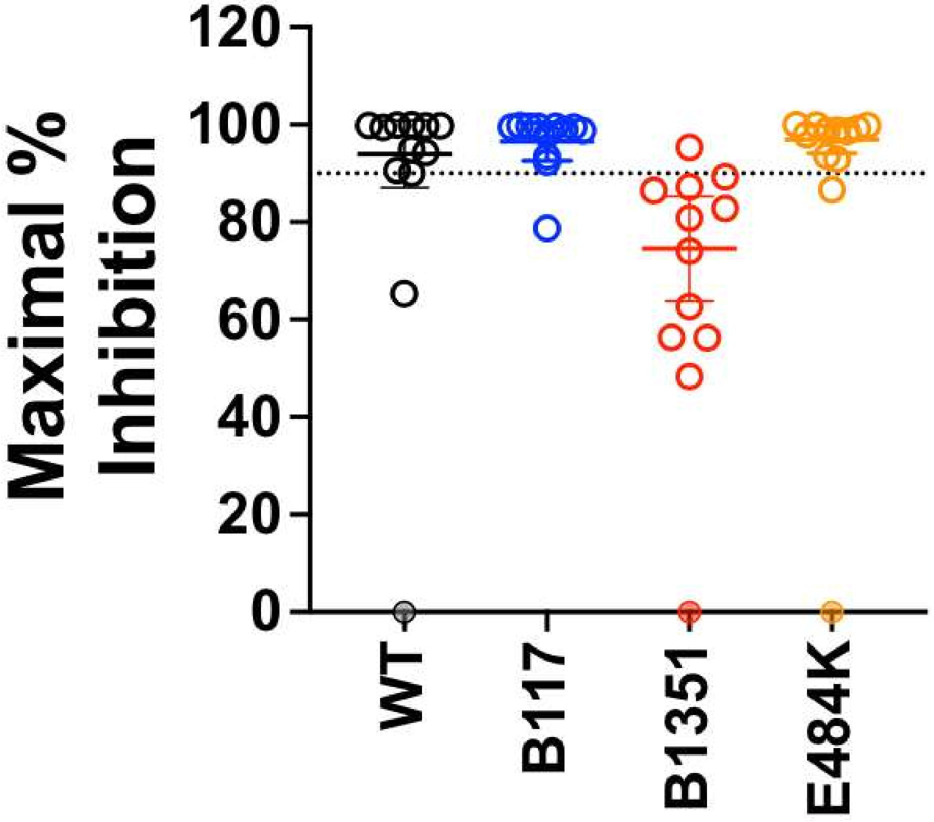

Extended Data Fig. S3. B.1.351 shows marked resistance to Sputnik vaccine sera neutralization. Maximal Percent Inhibition (MPI) at full serum strength extrapolated from nonlinear regression of log(inhibitor) versus normalized response, variable slope curve. Model used is from PRISM v9.0 where Y= 100/(1+10^((LogIC50-X)*HillSlope))). Log IC50 and Hill slope values were obtained for each curve generated in Extended Data Fig. S2. For reciprocal serum dilution of 1 (10^0), X= 0. Shaded colored circles at Y=0 for WT, B.1.351 and E484K represent data points from SP012 where no detectable neutralizing activity could be detected.
